# RNA interference-mediated silencing of G protein-coupled receptor 137 inhibits human gastric cancer cell growth

**DOI:** 10.3892/mmr.2014.3091

**Published:** 2014-12-15

**Authors:** ZISHU WANG, HUI ZHANG, JUNBIN WANG, YAN YANG, QIONG WU

**Affiliations:** 1 Department of Medical Oncology, The First Affiliated Hospital of Bengbu Medical College, Bengbu, Anhui 233004, P.R. China; 2 Department of Surgery Oncology, The First Affiliated Hospital of Bengbu Medical College, Bengbu, Anhui 233004, P.R. China

**Keywords:** G protein-coupled receptor 137, lentivirus, gastric cancer, tumorigenesis

## Abstract

G protein-coupled receptor 137 (GPR137) is an integral membrane protein, which belongs to the GPR137 family of cell surface mediators of signal transduction. GPF137 was recently identified; however, its role in human disease onset has remained to be elucidated. GPR137 is highly expressed in multiple human gastric cancer cell lines. A GPR137 short hairpin RNA (shRNA)-expressing vector was transfected into AGS and MGC80-3 gastric cancer cells, and the subsequent depletion of GPR137 resulted in a significant reduction in cell proliferation and colony formation, as determined by MTT and colony formation assays. In addition, cell cycle analysis indicated that GPR137 knockdown arrested MGC80-3 cells in G_2_/M phase. To the best of our knowledge, the present study was the first to investigate the role of GPR137 in gastric tumorigenesis and revealed that knockdown of GPR137 by lentivirus-mediated shRNA transfection inhibited the growth of gastric cancer cells *in vitro*. These results indicated that GPR137 may present a novel target for the development of pharmacological therapeutics for human gastric cancer.

## Introduction

Gastric cancer is a common malignancy on mainland China, with high rates of incidence and mortality (1). The diagnosis of novel gastric cancer cases in China comprise 40% of cases diagnosed worldwide every year (2,3). In addition, >80% of patients with gastric cancer in developing countries, including China, are diagnosed at advanced stages of the disease, resulting in poor prognoses (4,5). Despite major advances in gastric cancer research over the past few decades, an effective gastric cancer therapy has remained to be developed (6). However, a common feature of gastric cancer is an uncontrolled cell growth and proliferation mechanism. The identification of novel molecules associated with gastric cancer cell proliferation may aid the development of more effective therapies. The present study therefore aimed to identify additional molecules crucially involved in gastric cancer cell growth.

A diverse array of G protein-coupled receptors (GPRs; also known as GPCRs) with numerous functions have been implicated in the pathophysiology of multiple types of cancer and are under investigation as potential drug targets (7–9). GPRs are critical mediators of signal transduction and are involved in modulating the cellular proliferation of diverse types of cancer. Certain GPRs, including lysophosphatidic acid, acetylcholine, prostaglandin E2 and bradykinin receptors, induce the growth and proliferation of head and neck squamous-cell carcinomas (10,11). Proteolytic activation of protease-activated receptors (PARs, a GPR subtype) by thrombin stimulates the proliferation of breast carcinoma cells via the activation of epidermal growth factor signaling (12). Breast and ovarian cancer cell proliferation was also reported to be associated with endothelin A subtype receptor (ETAR, a GPR) (13), highlighting the prominent role of GPRs in cancer cell proliferation and growth.

G protein-coupled receptor 137 (GPR137), also known as transmembrane 7 superfamily member 1-like 1 protein (TM7SF1L1), C11orf4 or GPR-137A, and is a 417 amino acid member of the GPR-137 family of membrane proteins. The GPR137 family comprises three alternatively spliced isoforms: GPR137A (GPR137), GPR137B and GPR137C. The GPR137B isoform has been studied extensively and is upregulated in the kidney during development (14). GPR137B was also found to be abundant in the liver and heart but little or no expression was detected in the intestine, spleen or other tissues in the rat (15). Unlike GPR137B, GPR137 and GPR137C have rarely been studied. GPR137 was identified in 2003 by searching the GenBank genomic databases (16). Northern blot analysis revealed that GPR137 was detected in the hippocampus, but not in the hypothalamus, midbrain, thalamus, pons or basal forebrain. The role of GPR137 in human cancers is poorly understood, and the function of GPR137 and its regulatory mechanism in human cancer has remained to be elucidated.

Gene knockdown using small interfering RNA (siRNA) represents a useful tool for the assessment of the functional importance of cancer-associated genes *in vitro*. The present study therefore aimed to investigate the role of GPR137 in gastric cancer cell growth using RNA interference (RNAi).

## Materials and methods

### Cell lines and cell culture

The gastric cancer cell lines MKN28, SGC-7901, MGC80-3 and AGS were obtained from the American Type Culture Collection (Manassas, VA, USA). Cells were maintained in RPMI-1640 supplemented with 10% heat-inactivated fetal bovine serum (FBS) and penicillin/streptomycin (All from Gibco-BRL, Invitrogen Life Technologies, Carlsbad, CA, USA) at 37°C in a humidified atmosphere of 5% CO_2_.

### RNA extraction and reverse transcription quantitative polymerase chain reaction (RT-qPCR)

Total RNAs of cultured cells were extracted using TRIzol solution (Invitrogen Life Technologies, Carlsbad, CA, USA) 24 h following treatment. The cDNA was immediately reverse transcribed from isolated RNA using the SuperScript III First-Strand Synthesis System (Invitrogen Life Technologies). GPR137 mRNA expression was evaluated by qPCR analysis using the SYBR Premix Ex Taq™ Perfect Real-Time (Takara Bio, Inc., Otsu, Japan) on an ABI Prism 7500 real-time system (Applied Biosystems, Life Technologies, Foster City, CA, USA). GAPDH was used as the input reference. The primers used were as follows: GPR137 forward, 5′-ACCTGGGGAACAAAGGCTAC-3′ and reverse, 5′-TAGGACCGAGAGGCAAAGAC-3′; GAPDH forward, 5′-GTGGACATCCGCAAAGAC-3′ and reverse, 5′-AAAGGGTGTAACGCAACTA-3′.

Relative mRNA was determined using the formula 2^−ΔΔCT^ (with CT being the cycle threshold), where ΔCT = [CT (target gene) - CT (GAPDH)] as described previously (17).

### Construction of GPR137 short hairpin RNA (shRNA)-expressing lentivirus

To construct cell lines stably expressing GPR137 shRNA, an shRNA (5′-GAACAAAGGCTA CCTGGTATTCTCGAGAATACCAGGTAGCCTTTGTTCT TTTTT-3′) was inserted into the pFH-L plasmid (Shanghai Hollybio, Shanghai, China). A non-silencing siRNA (5′-TTCTCCGAACGTGTCACGT-3′) was used as a control. The lentivirus-based shRNA-expressing vectors were constructed, confirmed by DNA sequencing and named pFH-L-shGPR137 and pFH-L-shCon. For the transfection assay, AGS and MGC80-3 cells (5×10^4^ cells/well) were seeded into six-well plates and cultured for 72 h to reach 90% confluence. At 2 h prior to transfection, the culture medium was replaced with FBS-free RPMI-1640. The plasmid mixture containing pFH-L-shGPR137 (or pFH-L-shCon) and the pVSVG-I/pCMVΔR8.92 packaging vectors (Shanghai Hollybio), as well as Lipofectamine^®^ 2000 (Invitrogen Life Technologies) was added to the cells. Five hours following incubation, the culture medium was replaced with RPMI-1640 containing 10% FBS. The lentiviral constructs (Lv-shGPR137 or Lv-shCon) were harvested 48 h following transfection and purified by ultra-centrifugation as previously described (18,19). The lentivirus contained green fluorescent protein (GFP), and therefore the viral titer was determined by counting the number of GFP-expressing cells under an Olympus BX50 Brightfield/Fluorescence microscope (Olympus Corp., Tokyo, Japan) 96 h following infection, as previously described (20).

### Western blot analysis

Samples were homogenized in western blot analysis buffer containing 10 mM Tris-HCl (pH 7.4), 150 mM NaCl, 1% (v/v) Triton X-100, 1% sodium deoxycholate, 0.1% SDS, 5 mM EDTA, 1 mM phenylmethanesulfonylfluoride, 0.28 kU/l aprotinin, 50 mg/l leupeptin, 1 mM benzamidine, and 7 mg/l pepstain A (all purchased from Sigma-Aldrich, St. Louis, MO, USA). The homogenate was subsequently centrifuged at 12,000 × g for 15 min at 4°C and the supernatant was retained and preserved at −80°C for later use. The protein concentration was determined using a bicinchoninic acid assay kit (Pierce Biotechnology, Inc., Thermo Fisher Scientific, Rockford, IL, USA) and each sample was subjected to 10% SDS-PAGE. Proteins were transferred onto nitrocellulose membranes (EMD Millipore, Billerica, MA, USA) on a semi-dry electrotransferring unit (Bio-Rad Laboratories, Hercules, CA, USA), and incubated with rabbit anti-GPR137 polyclonal antibodies (11929-1-AP; 1:500; Proteintech Group Inc., Chicago, IL, USA) in Tris-buffered saline containing 0.1% Tween-20 (TBST; Amresco, Solon, OH, USA) and 5% non-fat dry milk overnight at 4°C. Following overnight incubation with the primary antibodies, membranes were washed with TBST and incubated with horseradish peroxidase-conjugated goat anti-rabbit and goat anti-mouse antibodies (sc-2054 and sc-2005; 1:5,000; Santa Cruz Biotechnology, Inc., Dallas, TX, USA) in TBST for 3 h. Immunoreactivity was detected using enhanced chemiluminescent autoradiography (ECL kit; GE Healthcare Life Sciences, Little Chalfont, UK). The membranes were re-probed with mouse anti-GAPDH monoclonal antibodies (sc-365062; 1:3,000; Santa Cruz Biotechnology, Inc.) following stripping.

### MTT assay

Cell proliferation was determined using a colorimetric assay with MTT. The MTT assay measures the conversion of MTT to insoluble formazan by the dehydrogenase enzymes of the intact mitochondria of living cells. Following treatment with or without the appropriate plasmid, AGS and MGC80-3 cells were seeded in 96-well plates (10^4^ cells/well). Cells were allowed to attach overnight and cell proliferation was evaluated over five days by measuring the conversion of MTT to formazan crystals. Briefly, 10 μl MTT reagent [5 mg/ml in phosphate-buffered saline (PBS); Sigma-Aldrich] was added to the cells and incubated for 4 h at 37°C. The medium was removed and 200 μl isopropanol was added. The amount of formazan crystals formed directly correlated with the number of viable cells. The reaction product was quantified by measuring the absorbance at 595 nm using an ELISA plate reader (Model 2550; Bio-Rad Laboratories, Inc). Experiments were performed in triplicate.

### Plate colony formation assay

Cells (10^4^ cells/well) were seeded into six-well plates three days following lentivirus infection. The medium was replaced at two-day intervals. Following six days of culture at 37°C, cells were washed with PBS and fixed with 4% paraformaldehyde (Sigma-Aldrich) for 30 min at room temperature. The fixed cells were then stained with freshly prepared diluted Giemsa (Merck KGaA, Darmstadt, Germany) for 10 min, washed with water and air-dried. The total number of colonies with >50 cells was counted using a Olympus BX50 Brightfield/Fluorescence microscope and an Olympus CH-2 Binocular Light microscope (Olympus Corp.).

### Fluorescence-activated cell sorting (FACS) analysis

The DNA contents of each cell cycle phase is reflected by variations in propidium iodide (PI) fluorescence intensities. The cell cycle distribution of Lv-shGPR137- or Lv-shCon-infected cells was analyzed by flow cytometry following PI staining as previously described (21). In brief, cells were collected 96 h after lentiviral infection and seeded into six-well plates (2×10^5^ cells/well). Cells were allowed to attach overnight prior to collection. Following washing with ice-cold PBS, cells were suspended in ~0.5 ml 70% cold ethanol and kept at 4°C for 30 min. The cells were subsequently treated with 100 mg/ml DNase-free RNase (Sigma-Aldrich) and incubated for 30 min at 37°C prior to the addition of PI (50 mg/ml; Sigma-Aldrich) directly to the cell suspension. The suspension was filtered through a 50-mm nylon mesh, and a total of 10,000 stained cells were subjected to flow cytometric analysis (FACSCalibur; BD Biosciences, San Jose, CA, USA).

### Statistical analysis

Data were analyzed using GraphPad Prism software version 6.00 for Windows (GraphPad Software, Inc., San Diego, CA, USA). Values are expressed as the mean ± standard deviation. Statistical significance between different groups was determined by repeated-measure analysis of variance. P<0.05 was considered to indicate a statistically significant difference between values.

## Results

### GPR137 is differentially expressed amongst gastric cancer cell lines

**Figure 1:**
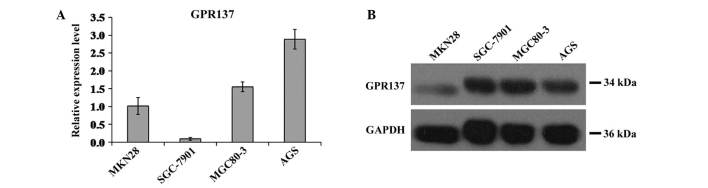
Expression profile of GPR137 in four gastric cancer cell lines. (A) GPR137 messenger RNA expression levels in four gastric cancer cell lines. (B) GPR137 protein expression levels in four gastric cancer cell lines. Values are presented as the mean ± standard deviation (n=3). GPR137, G protein-coupled receptor 137.

In order to investigate the role of GRP137 in gastric cancer, the expression levels of GPR137 were assessed in a subset of gastric cancer cell lines, including MKN28, SGC-7901, MGC80-3 and AGS. When normalized to the expression levels of GRP137 in MKN28 cells, GPR137 was observed to be differentially expressed amongst the four cell lines. The mRNA expression of GPR137 was highest in AGS cells, followed by MGC80-3 cells, and the lowest GPR137 mRNA expression levels were observed in SGC-7901 cells (Fig. 1A). As shown in Fig. 1B, western blot analysis revealed analogous results, indicating that GPR137 protein was expressed at high levels in MGC80-3 and AGS cells and at lower levels in MKN28 cells. mRNA levels of GPR137 in SGC-7901 cells were relatively low (Fig. 1A); however, the protein levels of GPR137 were markedly higher. These results revealed the differential expression profile of GPR137 amongst gastric cancer cell lines.

### Expression of GPR137 is suppressed by infection with Lv-shGPR137 in gastric cancer cells

**Figure 2:**
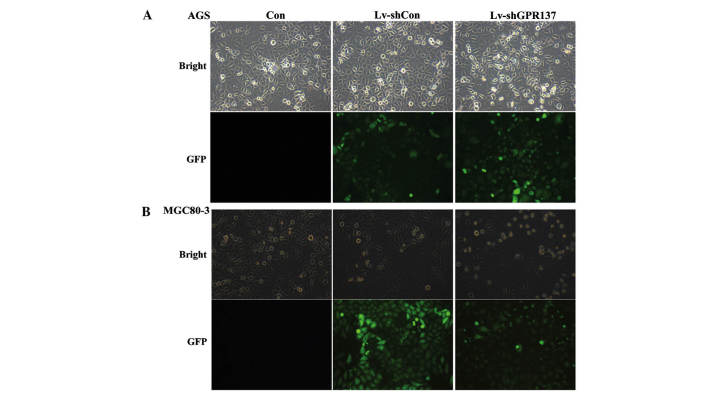
Determination of infection efficiency in human gastric cancer cells. Representative images of (A) AGS cells and (B) MGC80-3 cells following 96 h of lentivirus infection are shown (magnification, ×100). GPR137, G protein-coupled receptor 137; GFP, green fluorescent protein; Con, control; Lv-shCon, control lentivirus; Lv-shGPR137, GPR137 short hairpin RNA-expressing lentivirus.

**Figure 3:**
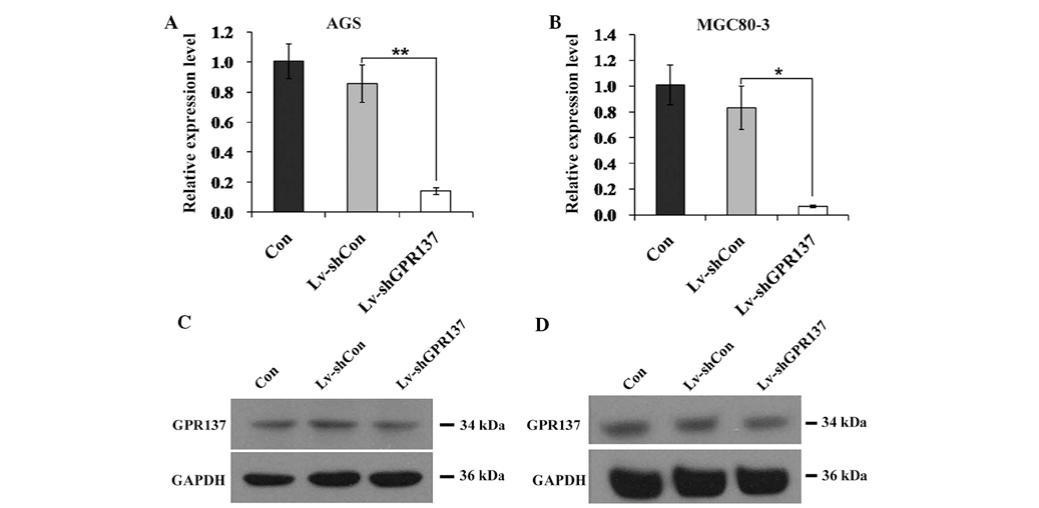
Expression of GPR137 is suppressed by infection with Lv-shGPR137. Analysis of GPR137 messenger RNA expression levels in (A) AGS cells and (B) MGC80-3 cells by reverse transcription-quantitative polymerase chain reaction in the non-infected, Lv-shCon and Lv-shGPR137 groups. GAPDH gene expression was used as the internal control. Analysis of GPR137 protein expression levels in (C) AGS cells and (D) MGC80-3 cells by western blot analysis in the non-infected, Lv-shCon and Lv-shGPR137 groups. GAPDH was used as a loading control. Values are presented as the mean ± standard deviation (n=3). ^*^P<0.05, ^**^P<0.01 vs. Lv-shCon. GPR137, G protein-coupled receptor 137; Con, control; Lv-shCon, control lentivirus; Lv-shGPR137, GPR137 short hairpin RNA-expressing lentivirus.

Due to the higher expression levels of GPR137 in MGC80-3 and AGS cells, and these cell lines were selected for subsequent analysis. To suppress the expression of GPR137 in gastric cancer cells, a lentivirus that stably expressed GPR137-specific siRNA (Lv-shGPR137) was constructed and infected into the two cell lines (multiplicity of infection, 20). Infection efficiency was ~90% in the two cell lines (Fig. 2). Furthermore, compared with the control vector Lv-shCon, the positive infection rate and the GPR137 mRNA expression levels in Lv-shGPR137-infected AGS cells were significantly decreased (P<0.01; Fig. 3A). Analogous results were obtained for MGC80-3 cells (P<0.05; Fig. 3B). Concomitantly, the protein expression levels of GPR137 were markedly reduced in AGS and MGC80-3 cells following infection with Lv-shGPR137 (Fig. 3C and D). The expression of GPR137 was therefore successfully inhibited by infection with Lv-shGPR137 in gastric cancer cells.

### Knockdown of GPR137 inhibits gastric cancer cell growth

**Figure 4:**
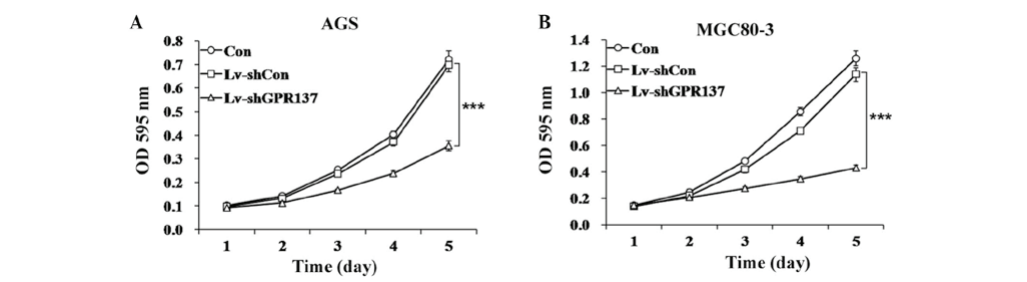
GPR137 knockdown suppresses the proliferation of gastric cancer cells. The proliferation of (A) AGS cells and (B) MGC80-3 cells in the Lv-shGPR137 groups was significantly inhibited, as detected by MTT assay. Values are presented as the mean ± standard deviation (n=3). ^***^P<0.001 vs. Lv-shCon. GPR137, G protein-coupled receptor 137; Con, control; Lv-shCon, control lentivirus; Lv-shGPR137; GPR137 short hairpin RNA-expressing lentivirus; OD, optical density.

**Figure 5:**
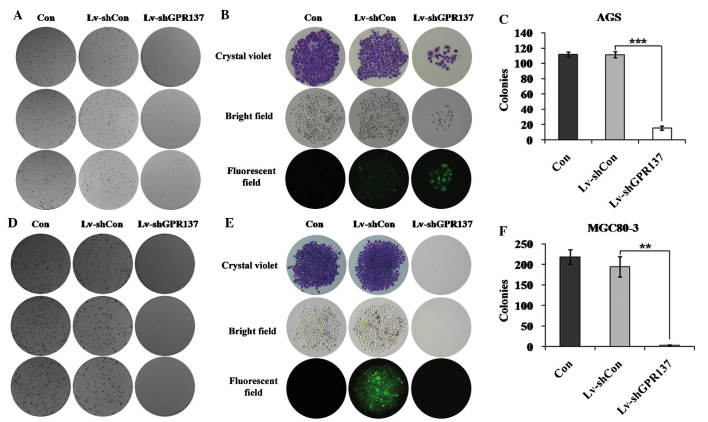
Knockdown of GPR137 inhibits the colony-forming ability of gastric cancer cells. Representative images of colonies in (A and B) AGS cells and (D and E) MGC80-3 cells under light and fluorescence microscopy (magnification, ×40). Statistical analysis of the number of colonies in (C) AGS cells and (F) MGC80-3 cells stained with crystal violet staining are also presented. Values are presented as the mean ± standard deviation (n=3). ^**^P<0.01, ^***^P<0.001 vs. Lv-shCon. GPR137, G protein-coupled receptor 137; Con, control; Lv-shCon, control lentivirus; Lv-shGPR137, GPR137 short hairpin RNA-expressing lentivirus.

To investigate the role of GPR137 in gastric cancer cell growth, cell viability and colony formation assays were conducted. MGC80-3 and AGS cells were infected with Lv-shGPR137 or Lv-shCon. In the cell viability assay, cell numbers were evaluated on days one to five of culture. The number of cells in the control and Lv-shCon groups was similar, whereas cell proliferation was markedly reduced in AGS cells infected with Lv-shGPR137 (P<0.001; Fig. 4A). The proliferation rate of MGC80-3 cells was also decreased following GPR137 knockdown (P<0.001; Fig. 4B). On day five, the proliferation rate of cells in the Lv-shGPR137 groups was markedly reduced, by 49.2% in AGS cells and 62.2% in MGC80-3 cells, compared with that of the Lv-shCon groups. The colony formation assay revealed that AGS cells failed to form as many colonies as the control and Lv-shCon groups following infection with Lv-shGPR137 (Fig. 5A and B). A mean of 15 colonies were generated in Lv-shGPR137-infected AGS cells, compared with a mean of 112 colonies in the control group and 111 colonies in the Lv-shCon group (P<0.001; Fig. 5C). The number of colonies was also reduced in MGC80-3 cells following GPR137 knockdown (Fig. 5D and E). Almost no colonies were observed in Lv-shGPR137-infected MGC80-3 cells compared with 218 colonies in the control group and 194 colonies in the Lv-shCon group (P<0.01; Fig. 5F). These results indicated that knockdown of GPR137 decreased gastric cancer cell proliferation.

### GPR137 depletion arrests cell cycle progression in gastric cancer cells

**Figure 6:**
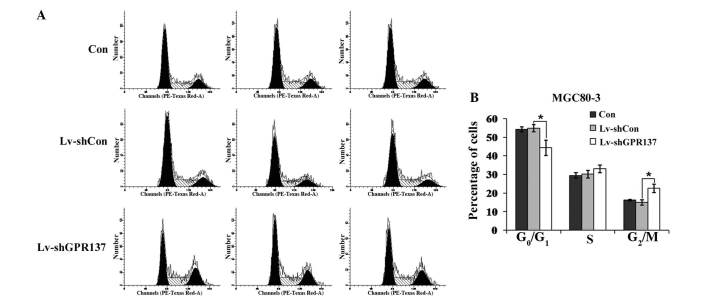
Knockdown of GPR137 arrests the cell cycle of MGC80-3 cells. (A) Cell cycle distribution in the Con, Lv-shCon and Lv-shGPR137 groups, evaluated by flow cytometry. (B) Cell percentage in distinct cell cycle stages varied between each group. Values are presented as the mean ± standard deviation (n=3). ^*^P<0.05 vs. Lv-shCon. GPR137, G protein-coupled receptor 137; Con, control; Lv-shCon, control lentivirus; Lv-shGPR137; GPR137 short hairpin RNA-expressing lentivirus.

To assess the effects of GPR137 depletion on cell cycle distribution, MGC80-3 cells infected with Lv-shCon or Lv-shGPR137 were synchronized by serum starvation for 72 h. The culture medium was subsequently replaced with complete medium containing 10% FBS and cell cycle progression was assayed 24 h later by flow cytometry. The number of MGC80-3 cells at each phase (G_0_/G_1_, S or G_2_/M) was differentially distributed amongst the three groups (Con, Lv-shCon and Lv-shGPR137; Fig. 6A). At each phase, cells were similarly distributed amongst the non-infected and Lv-shCon-infected MGC80-3 cells (Fig. 6B). However, a greater proportion of cells were halted in G2/M phase (~22.6%) when GPR137 was depleted (P<0.05). The proportion of cells in G_0_/G_1_ phase was significantly reduced when GPR137 was depleted, accounting for only 44.41%, compared to 54.31% in the control and Lv-shCon groups (P<0.05). These results suggested that the cell cycle was arrested following GPR137 knockdown, which indicated that GPR137 may have a significant role in gastric cancer cell cycle progression.

## Discussion

The development of gastric carcinogenesis is complex and multifactorial, involving *Helicobacter pylori,* as well as environmental and genetic factors (22). The development of gastric cancer occurs following the accumulation of multiple genetic and epigenetic alterations during the lifetime of the individual in question (22). These changes eventually trigger extracellular signals to become intracellular signals. The majority of patients with gastric cancer present with late-stage disease and have poor prognoses; therefore, the identification of aberrantly expressed proteins associated with gastric carcinogenesis was required. GPR137 was previously identified as a novel G protein-coupled receptor and its transcript was detected in diverse brain tissues (16). However, the role of GPR137 in organs outside of the human brain remained to be elucidated. The results of the present study demonstrated that GPR137 was able to mediate gastric cancer cell growth *in vitro*. Depletion of GPR137 by lentivirus-delivered shRNA markedly reduced the number of cells detected using a cell viability assay. Concomitantly, gastric cancer cells failed to maintain colony formation ability following infection with Lv-shGPR137. In addition, cell cycle phase distribution was altered.

Cell viability assays were used to assess the cell proliferation rate which also gave an indication of cell numbers (23). Similarly, colony formation assays facilitated the assessment of cell growth capability under anchorage-independent conditions, and are closely associated with the *in vivo* situation (24). The observed attenuation of colony formation indicated that GPR137 knockdown impaired the anchorage-independent growth of gastric cancer cells. To the best of our knowledge, the present study was the first to report that GPR137 has a key role in mediating cancer cell growth.

Elucidating the role of GPR137 in gastric cancer cell growth is of biological significance. GPRs have been implicated in the mediation of cellular proliferation in diverse types of cancer. However, GPR137 was initially identified as a GPR in 2003 and its mRNA was only detected in brain tissues (16). Whether GPR137 functions as a key mediator of cancer growth in the same way as other GPRs do has remained elusive. The present study identified a novel function of GPR137 to gastric tumorigenesis, and may provide a target for the development of gastric cancer therapeutics.

Of note, GPR137B was previously implicated in kidney development (14). GPR137B was also identified to be a novel lysosome integral membrane protein and performed certain lysosome functions, including autophagy, degrading of products and transport of nutrition (15). However, GPR137 potentially regulates gastric cancer cell growth via alternative mechanisms, rather than lysosome signaling pathways. The hypothesis underlying the present study was based upon the observation that the GPR137 transcript was detected in brain tissues, indicating that certain ligands secreted by the brain may function as stimuli of GPR137. The results of the present study demonstrated the tumor promoting-effect of GPR137 in the stomach. It was therefore suggested that specific ligands existing in the stomach and brain may be involved in GPR137-mediated gastric cancer cell growth. A further potential mechanism underlying the tumor-promoting effect of GPR137 is that GPR137 may regulate molecules which are involved in cell cycle regulation. GPR137 depletion led to abnormal accumulation of MGC80-3 cells in the S phase and, in particular, the G_2_/M phase. GPR137 may therefore contribute to gastric cancer cell growth via manipulation of G_2_/M phase regulators. A typical example of cell cycle manipulation is that the knockdown of aurora kinase A induces G_2_/M phase accumulation via the regulation of bipolar spindle formation (25). Therefore, it was hypothesized that GPR137 may regulate the cell cycle by influencing G_2_/M phase molecules, which mediate microtubule and/or spindle activities, and in this way promote gastric cancer cell growth. Further study is required in order to investigate the validity of this hypothesis.

In conclusion, to the best of our knowledge, the present study was the first to define GPR137 as a functional mediator of gastric cancer cell growth. Knockdown of GPR137 significantly inhibited gastric cancer cell growth *in vitro*. These results may aid the development of novel gastric cancer drug therapeutics.
